# First detection and molecular characterization of three trichomonad species in experimental beagle dogs in China

**DOI:** 10.1051/parasite/2026056

**Published:** 2026-10-02

**Authors:** Xiao-Long Liao, Xue-Li Ge, Rong-Jun Gong, Jia-Min Ma, Yong-Hao Wang, Li-Qin Gui, Qing-Hong Guo, Xin-Chao Liu, Wen-Chao Li

**Affiliations:** Anhui Province Key Laboratory of Animal Nutritional Regulation and Health, College of Animal Science, Anhui Science and Technology University Fengyang 233100 PR China

**Keywords:** Prevalence, *Pentatrichomonas hominis*, *Tritrichomonas foetus*, *Dientamoeba fragilis*, Experimental beagle dogs, China

## Abstract

Three different parasites of the phylum Parabasalia (*Pentatrichomonas hominis, Tritrichomonas foetus* and *Dientamoeba fragilis*) have been described in dogs. However, limited information about the prevalence of these trichomonad species in experimental beagle dogs in China is available. The aim of the current study was to investigate the frequency and molecular characterization of these three parasites species in these dogs. A total of 452 fecal samples were collected from ordinary clean-grade experimental beagle dogs and examined using PCR analysis, and positive samples were subsequently sequenced for species identification and analysis. Results showed that the prevalence of *P. hominis*, *T. foetus* and *D. fragilis* in the experimental dogs was 6.4% (29/452, 95% CI: 4.1–8.7%), 0% (0/452) and 0.7% (3/452, 95% CI: 0–1.4%), respectively. The sequence and phylogenetic analyses showed that the obtained sequences of *P. hominis* SSU rRNA genes were similar to the previously reported genotype CC1, and the sequences of *D. fragilis* SSU rRNA genes belonged to genotype 1, which is the most common genotype in humans. Our study provides the first data on the molecular prevalence of *P. hominis*, *T. foetus* and *D. fragilis* in experimental beagle dogs globally.

## Abstract (fr)

Trois parasites différents appartenant à l'embranchement des Parabasalia (*Pentatrichomonas hominis*, *Tritrichomonas foetus* et *Dientamoeba fragilis*) ont été décrits chez le chien. Toutefois, les informations concernant la prévalence de ces espèces de trichomonades chez les chiens de race Beagle utilisés pour l'expérimentation en Chine sont limitées. L'objectif de la présente étude était d'examiner la fréquence et la caractérisation moléculaire de ces trois espèces de parasites chez ces chiens. Au total, 452 échantillons de selles ont été prélevés sur des chiens de race Beagle élevés en conditions expérimentales standard (niveau de propreté courant) et analysés par PCR et les échantillons positifs ont ensuite été séquencés à des fins d'identification et d'analyse. Les résultats ont montré que la prévalence de *P. hominis*, *T. foetus* et *D. fragilis* chez les chiens de l'étude était respectivement de 6,4 % (29/452 ; IC à 95 % : 4,1–8,7 %), 0 % (0/452) et 0,7 % (3/452 ; IC à 95 % : 0–1,4 %). Les analyses de séquences et les analyses phylogénétiques ont révélé que les séquences obtenues pour les gènes de l'ARNr SSU de *P. hominis* étaient similaires au génotype CC1 décrit précédemment, tandis que la séquence des gènes de l'ARNr SSU de *D. fragilis* appartenait au génotype 1, qui est le génotype le plus répandu chez l'homme. Notre étude fournit les premières données mondiales sur la prévalence moléculaire de *P. hominis*, *T. foetus* et *D. fragilis* chez des chiens de race Beagle utilisés pour l'expérimentation.

## Introduction

Trichomonads are anaerobic, flagellated protists, including both pathogenic species and some symbiotic species. Certain species colonize the oral, digestive and urogenital tracts of vertebrate and invertebrate hosts, with at least five species recognized in dogs: *Pentatrichomonas hominis* (Davaine, 1860) Wenrich, 1931, *Tritrichomonas foetus* (Riedmüller, 1928) Wenrich & Emmerson, 1933, *Trichomonas tenax* (Muller, 1773) Dobell, 1939, *Trichomonas brixi* Kellerová & Tachezy, 2017 and *Dientamoeba fragilis* Jepps & Dobell, 1918 [4, 19, 23, 25].

*Pentatrichomonas hominis*, formerly known as *Trichomonas hominis* Davaine, 1860, is an identified pathogenic zoonotic protozoan parasitizing the cecum and colon of humans and other mammals. It has been associated with diarrhea in humans and various animal hosts, as well as pulmonary infections and rheumatoid arthritis in human [5, 21, 34–35]. *Tritrichomonas foetus* is an intriguing protozoan parasite that infects multiple animal hosts, primarily cattle and cats, with occasional isolation from pigs and dogs [6, 8]. It causes bovine trichomoniasis, a sexually transmitted disease mainly leading to infertility and abortion in cattle, and feline trichomoniasis, a newly emerging gastrointestinal disorder that results in chronic large bowel diarrhea in felines [6, 7]. *Trichomonas tenax* and *Trichomonas brixi* are zoonotic flagellated protozoans identified in the oral cavities of humans and animals and have been associated with periodontal disease [19]. *Dientamoeba fragilis* is one of the most common parasites of the human bowel worldwide, with amoeba-like morphology [33]. Following morphological and molecular studies, this parasite was recognized phylogenetically as a flagellated trichomonad [1, 3, 29]. *Dientamoeba fragilis* has been detected in humans, non-human primates, pigs, cats, dogs, rats, rabbits, pet budgerigars, and arthropod vectors, and the possibility of zoonotic transmission between humans and animals has been recognized [9–11, 18, 32–33].

Beagle dogs are an internationally recognized standard experimental breed widely used in medicine, biology, pathology, oncology and other life sciences [20]. With the rapid development of China’s biomedical industry, the demand for experimental beagles has increased significantly, and domestic related breeding has developed rapidly. During the breeding process of beagle dogs, parasitic infections not only pose a serious threat to the health of experimental beagles, but also significantly affect the accuracy of scientific research data. So far, no epidemiologic data regarding the presence of the above mentioned trichomonad species in experimental beagle dogs in China are available. The aim of this study was to investigate the prevalence and molecular characterization of three trichomonad species in experimental beagle dogs in China.

## Materials and methods

### Collection of fecal samples

A total of 452 fresh fecal samples of ordinary clean-grade beagle dogs were collected from experimental beagle dog farms in Beijing (150), Lianyungang (84) and Nantong (218), between March to May 2025. Each fecal specimen was collected in an individual disposable plastic pouch, and stored at −20 °C until DNA extraction. Because fresh fecal droppings were collected from the cages of dogs (not from the animals directly), no animal ethics approval was required in this study.

### DNA extraction and PCR amplification

Genomic DNA was extracted from approximately 200 mg of each fecal sample with an E.Z.N.A^®^ Mag-Bind^®^ Stool DNA Kit (Omega Bio-tek, Norcross, GA, USA), according to the manufacturer’s instructions. The final nucleic acids were stored at −20 °C.

Three trichomonad species in samples were amplified by nested PCR assays of specific SSU rRNA gene regions using the primer pairs and detailed protocols listed in Table 1, according to the nested PCRs previously described by Li *et al.*, 2015 [22], Vandenberg *et al.*, 2006 [31], and Cacciò *et al.*, 2012 [2], respectively. The first round of PCR used the extracted fecal genomic DNA as a template, and the second round of PCR used the first round of PCR product as a template. All the PCR analyses contained positive and negative controls (distilled water). The secondary PCR products were electrophoresed on a 1.5% agarose gel and visualized using a gel documentation system. Amplicons from pathogen-positive samples were sequenced in both directions with corresponding amplification primers (Sangon Biotech, Shanghai, China).

**Table 1:** Primers and PCR procedure for three trichomonad species in this study.

Primers		Sequences (5′–3′)	PCR procedure	Size (bp)	Reference
First PCR for *P. hominis* and *T. foetus*	FF	GCGCCTGAGAGATAGCGACTA	95 °C for 10 min, then 30 cycles (95 °C for 60 s, 60 °C for 60 s, and 72 °C for 60 s), followed by 72 °C for 10 min		[22]
	RR	GGACCTGTTATTGCTACCCTCTTC			
Second PCR for *P. hominis*	hF	TGTAAACGATGCCGACAGAG	95 °C for 10 min, then 30 cycles (95 °C for 30 s, 55 °C for 30 s, and 72 °C for 30 s), followed by 72 °C for 10 min	339	
	hR	CAACACTGAAGCCAATGCGAGG			
Second PCR for *T. foetus*	sF	GGTTGTTTGTATAGGATTGC	95 °C for 10 min, then 30 cycles (95 °C for 30 s, 54 °C for 30 s, and 72 °C for 30 s), followed by 72 °C for 10 min	452	
	sR	TGCCCTCATAAAAGGACAA			
First PCR for *D. fragilis*	DF1	CTCATAATCTACTTGGAACCAATT	95 °C for 4 min, followed by 40 cycles, each consisting of 95 °C for 30 s, 58 °C for 30 s, 72 °C for 1 min, and a final extension at 72 °C for 10 min		[31]
	DF4	CCCCGATTATTCTCTTTGATATT			
Second PCR for *D. fragilis*	DF322For	GAGAAGGCGCCTGAGAGATA	95 °C for 4 min, followed by 40 cycles, each consisting of 95 °C for 30 s, 54 °C for 30 s, 72 °C for 1 min, and a final extension at 72 °C for 10 min	366	[2]
	DF687Rev	TTCATACTGCGCTAAATCATT			

### Sequence and phylogenetic analyses

The qualified forward and reverse raw sequences obtained in this study were assembled into a final consensus sequence using the *de novo* assembly plugin in DNAMAN 6.0 software. Final sequences were aligned with BioEdit7.1 (http://www.mbio.ncsu.edu/BioEdit/bioedit.html) and examined with BLAST searches (http://www.ncbi.nlm.nih.gov/BLAST). The phylogeny was inferred using a neighbor-joining (NJ) method with the Kimura 2-parameter model in MEGA 6.06 (http://www.megasoftware.net/), with 1,000 bootstrap replicates. Representative SSU rRNAs sequenced in this study have been deposited in GenBank under accession numbers PZ028360–PZ028361 for *P. hominis* and PZ028364 for *D. fragilis*.

## Results

### Frequency of *P. hominis*, *T. foetus* and *D. fragilis*

The specific PCR detected *P. hominis*, *T. foetus* and *D. fragilis* in 29, 0 and 7 samples out of 452 samples. Sanger sequencing confirmed the presence of *P. hominis* in all 29 PCR positive samples, but only 3 *D. fragilis* PCR positive samples were sequenced successfully. Only these verified samples were considered positive (Table 2). According to this, the overall prevalence of *P. hominis* and *D. fragilis* in the samples, as determined by PCR analysis, was 6.4% (29/452, 95% CI: 4.1–8.7%) and 0.7% (3/452, 95% CI: 0–1.4%), respectively (Table 2). No *T. foetus* infections were detected in this study. The remaining four *D. fragilis* PCR positive samples failed to sequence, so they were classified as negative.

**Table 2:** Prevalence of *P. hominis, T. foetus* and *D. fragilis* infections in experimental Beagle dogs in China.

Sampling site	No. samples	*P. hominis*		*T. foetus*		*D. fragilis*	
		No. positive	Positive rate/% (95% CI)	No. positive	Positive rate/% (95% CI)	No. positive	Positive rate/% (95% CI)
Beijing	150	6	4.0 (0.8–7.2)	0	0	1	0.7 (0–2.0)
Lian Yun Gang	84	2	2.4 (0–5.7)	0	0	0	0
Nan Tong	218	21	9.6 (5.7–13.6)	0	0	2	0.9 (0–2.2)
Total	452	29	6.4 (4.1–8.7)	0	0	3	0.7 (0–1.4)

The prevalence rates of *P. hominis* in beagle dogs in Beijing, Lianyungang and Nantong were 4.0% (6/150, 95% CI: 0.8–7.2%), 2.4% (2/84, 95% CI: 0–5.7%) and 9.6% (21/218, 95% CI: 5.7–13.6%), respectively. The prevalence rates of *D. fragilis* in beagle dogs in Beijing, Lianyungang and Nantong were 0.7% (1/150, 95% CI: 0–2.0%), 0.0% (0/84) and 0.9% (2/218, 95% CI: 0–2.2%), respectively. There were no coinfections with *P. hominis* and *D. fragilis* in the present study.

### Molecular analysis of *P. hominis* and *D. fragilis*

The 29 *P. hominis*-positive PCR amplicons were sequenced successfully, and two different sequences were obtained in the present study (Seq1, GenBank accession: PZ028360, *n* = 26; Seq2, GenBank accession: PZ028361, *n* = 3), and the two sequences differed at 1 and 2 nucleotides, compared with the previously reported genotypes CC1 (GenBank accession: KJ408929), respectively [24]. Phylogenetic analyses based on the NJ method also showed that the two sequences obtained in this study formed a monophyletic cluster with the *P. hominis* isolates from several animal hosts (Fig. 1).

**Figure 1:**
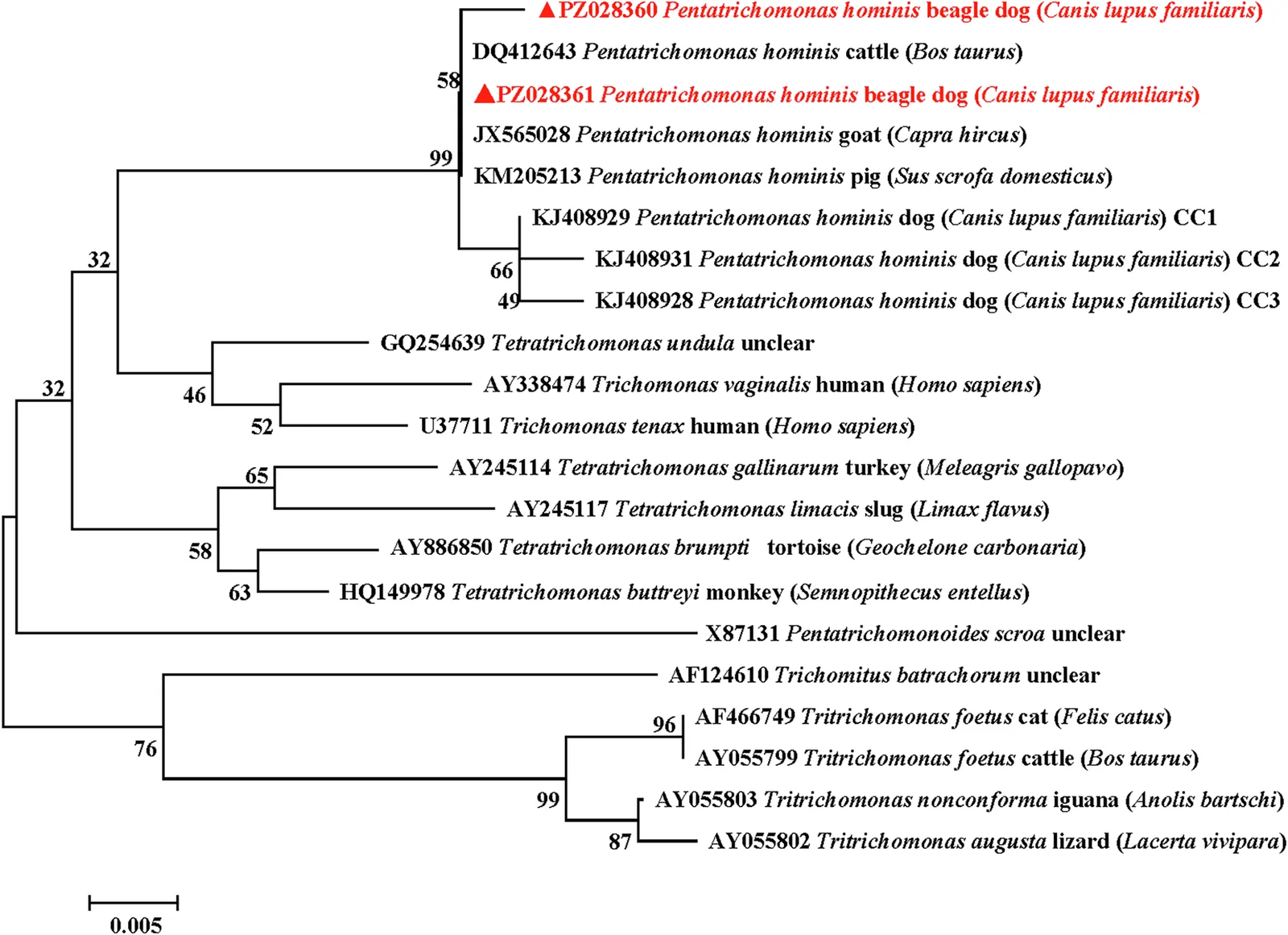
Neighbor-joining trees showing that the SSU rRNA sequences of *Pentatrichomonas hominis* obtained from the experimental beagle dogs in this study (indicated by solid red triangles) are located on the *P. hominis* branches.

Of a total of 7 *D. fragilis* PCR-positive samples, only 3 yielded successful sequencing results. Three isolates had identical sequences and represented a single isolate (GenBank accession: KJ408929). Blast analyses displayed whole identity with several isolates reported from humans and budgerigars. The NJ tree showed that the sequence obtained in this study formed a monophyletic cluster with the isolates of genotype 1 from humans and budgerigars (Fig. 2).

**Figure 2:**
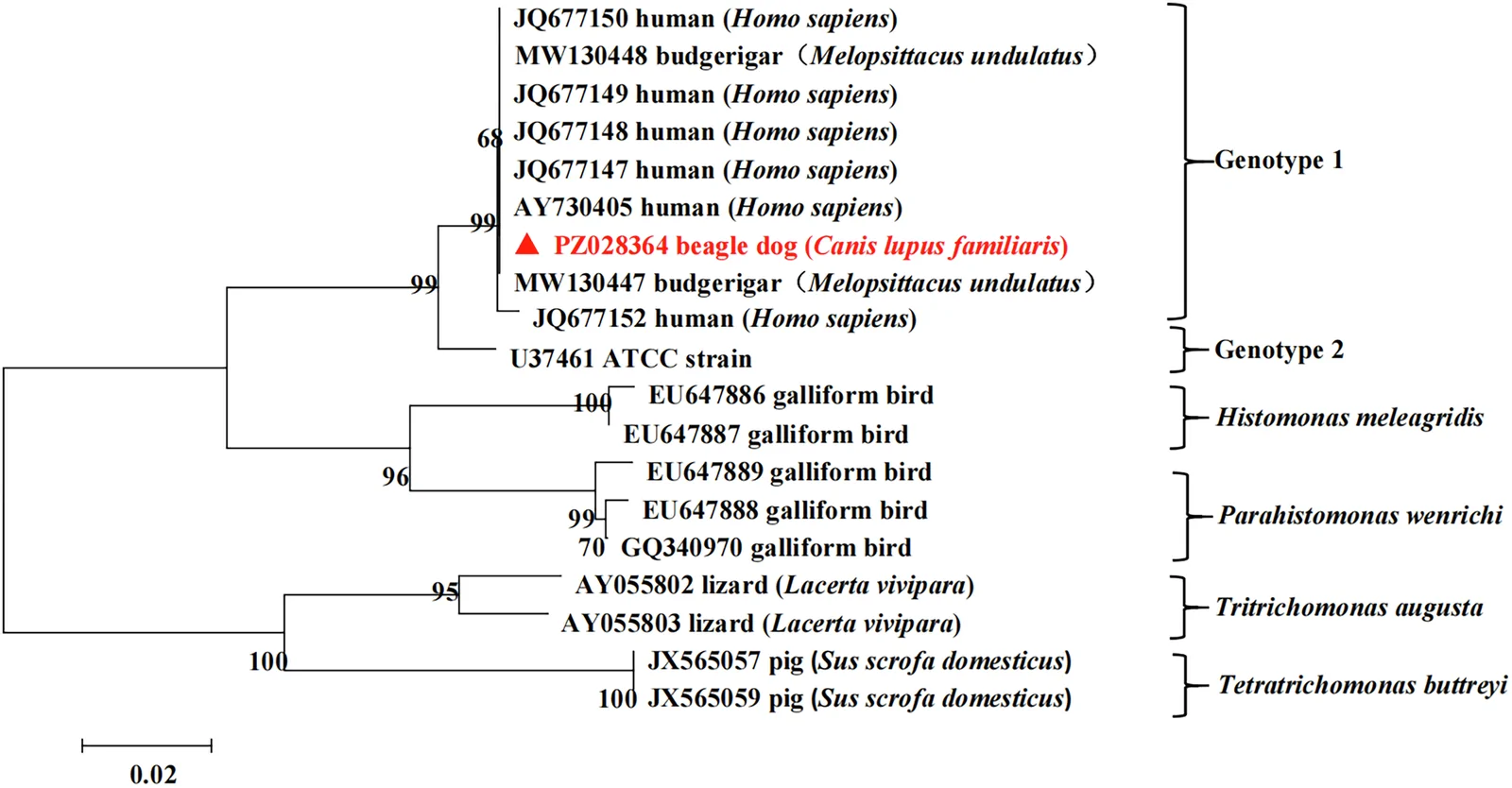
Phylogenetic analysis of the SSU rRNA sequences from different trichomonad isolates. The *Dientamoeba fragilis* isolate reported in this study is indicated with a solid red triangle.

## Discussion

In experimental animals, researchers pay more attention to the use of the animals in scientific research than to the diseases of the animals themselves. Knowledge on the presence of zoonotic pathogens in experimental beagles is limited, and as far as we know, there are currently no reports on trichomonad species in experimental beagles globally.

*Pentatrichomonas hominis* has been reported worldwide and can infect many animal hosts, especially with a high prevalence in pigs, cattle, goats, and dogs [26, 34]. The prevalence of *P. hominis* in experimental beagles in this study is 6.4% (29/452), which was lower than the reported rates of canine *P. hominis* in the northern (27.4%, 69/252), eastern (31.4%, 99/315) and southern (22.4%, 66/294) regions of China [17, 23–24]. Our observed levels are also lower than those found in France (15.8%, 34/215), Poland (12.2%, 5/41), and the United States (92.9%, 13/14), while our results are similar to those found in Japan (7.0%, 38/544) [13, 16, 27, 30].

*Tritrichomonas foetus* is a protozoan parasite that has been traditionally identified as a cause of reproductive tract disease in cattle and gastrointestinal tract infection in cats [6]. In recent years, *T. foetus* or *T. foetus*-like trichomonads have successively been isolated from the bodies of patients with pneumonia or acute respiratory syndrome, suggesting that *T. foetus* may possess a certain potential for zoonosis [25]. Since Gookin *et al.*, 2005 [12] first reported that dogs can be infected with *T. foetus*, molecular methods have indicated that the infection rate of *T. foetus* in dogs is 5.3% (2/38) in the United States, 0.6% (2/315) in eastern China and 5.8% (17/294) in southern China [17, 24, 30]. However, no *T. foetus* infections were detected in this study.

*Dientamoeba fragilis* is a neglected trichomonad in human and animal studies, even though it has been more than 100 years since its initial discovery [4]. Aside from data in humans, little information is available about the natural host range of *D. fragilis.* Sparse published data indicate that *D. fragilis* has been detected in a few animal species including rats, cats, dogs, cows, pigs, goats, rabbits, sheep, guinea pigs, budgerigars and non-human primates (Table 3). For dogs, only qPCR data from two recently published reports can be used, with infection rates of 1.8% (1/56) in Australia and 7.4% (4/54) in Czechia [4, 18]. Based on Sanger sequencing of the PCR products, the molecular prevalence of *D. fragilis* in ordinary clean-grade experimental beagle dogs in China was found to be 0.7% (3/452) in the present study.

**Table 3:** Molecular detection of *D. fragilis* in non-human hosts and foods in various studies.

Host	Country	Detection method	Positive rate/%	Reference
Gorilla	Australia	PCR	30.0 (3/10)	[28]
Pig	Italy	qPCR, PCR and sequencing	63.2 (24/38)	[2]
Dog	Australia	qPCR	1.8 (1/56)	[4]
Cat		qPCR	2.3 (1/43)	
Cattle	Türkiye	PCR	19.0 (31/163)	[33]
Budgerigar	Türkiye	qPCR	21.3 (32/150)	[32]
Dog	Czechia	qPCR	7.4 (4/54)	[18]
Cat		qPCR	5.3 (1/19)	
Horse		qPCR	13.3 (2/15)	
Rabbit		qPCR	38.5 (5/13)	
Sheep		qPCR	16.7 (1/6)	
Goat		qPCR	25.0 (1/4)	
Guinea pig		qPCR	50.0 (1/2)	
Rabbit		PCR	7.7 (1/13)	
Housefly	Türkiye	PCR	8.0 (12/150)	[10]
Cattle	Australia	qPCR	73.5 (36/49)	[14]
Raw Milk and Cheese	Türkiye	PCR	6.0 (9/150)	[11]

Compared to pet dogs, household dogs and farm dogs, the breeding of experimental dogs has strict environmental and management requirements, and the low infection rates by *P. hominis*, *T. foetus* and *D. fragilis* in the dogs in this study may be largely attributed to this.

The obtained sequences of the *P. hominis* SSU rRNA genes in this study exhibit minor allelic variations compared with the previously reported genotypes CC1 of *P. hominis*. This may involve expected variation within the multiple copies of the SSU rRNA genes in any given genome or may result from parasite-host or parasite-environment adaptation [23]. Currently, molecular analysis has demonstrated limited genetic variability within *D. fragilis*, and two genotypes, designated genotype 1 and genotype 2, have been described, with genotype 1 reported in a wide range of hosts, including both humans and animals [2]. Analysis of the SSU rRNA partial gene region showed that the sequence obtained in this study belongs to genotype 1 and has 100% identity with several isolates reported from humans and budgerigars, suggesting the potential for zoonotic transmission.

The published data, including the isolation of *P. hominis* and *T. foetus* strains from dogs and animal infection, have supported dogs as natural hosts for *P. hominis* and *T. foetus* [12, 21]. However, it is very difficult to draw the conclusion that dogs can act as a natural host of *D. fragilis* based on the previous and present research [4, 14]. The previous reports provided only qPCR data for *D. fragilis* in dogs, and the qPCR results generated by these assays need to be optimized and validated to confirm their accuracy, because reports have revealed that qPCR assays may cross-react with non-target species [4, 14]. Whether the three beagle dogs were infected naturally with *D. fragilis* or merely via mechanical passage of the cyst or pre-cyst stages of *D. fragilis* rather than indigenous infections remains uncertain. Moreover, the absence of clinical signs, the absence of trophozoites or cyst detection in feces, and the relatively low detection rate by PCR in this study further indicate that it is premature to conclude that beagle dogs are natural hosts to *D. fragilis.*

Currently, many characteristics of the three trichomonad species, especially *D. fragilis*, including the parasite’s biology, life cycle, host range, pathogenicity, possible transmission routes and genetic diversity etc., remain poorly understood at best, although some important advances have been made in recent years. The present study was, to our knowledge, the first molecular assessment of *P. hominis*, *T. foetus* and *D. fragilis* in experimental beagle dogs in China. However, this study has some limitations. First, the sample size and sampling regions are relatively limited, involving only three cities and 452 samples. Second, fecal samples from staff who have been in close contact with the experimental beagle dogs, as well as environmental samples, were not collected simultaneously and tested for the three trichomonad species, which makes it difficult to assess any potential zoonotic transmission of these trichomonad species. Finally, several published *D. fragilis* molecular assays, including the method employed in this study, cross react with other closely related trichomonads such as *T. foetus*, *P. hominis* and *T. mobilensis*, in animal stool specimens [4, 14]*.* This means that while these molecular methods can be used for the detection of *D. fragilis* in animal samples, additional evidence including microscopy or sequencing are required to confirm *D. fragilis* infection in a new host species [15]. However, the present study used only PCR and sequencing to analyse *D. fragilis*, and fecal microscopy was not applied here. This is also the reason why the remaining four *D. fragilis* PCR-positive samples that failed to be sequenced were not recognized as positive when calculating the observed infection rate. This highlights the urgent need to clarify zoonotic transmission of these trichomonad species by using large-scale sampling and different animal species, and to develop a rapid and sensitive molecular assay for trichomonad species, especially *D. fragilis*, in animal samples.

## Conclusion

Our findings provide insights into the potential zoonotic risks associated with *P. hominis*, *T. foetus* and *D. fragilis* in clean-grade experimental beagle dogs and highlight the need to improve the health and microbiological quality of experimental animals through comprehensive pathogen detection and integrated infection control.
